# Tatouage au henné noir: attention à l’eczéma de contact

**DOI:** 10.11604/pamj.2018.30.46.15563

**Published:** 2018-05-18

**Authors:** Ouiam El Anzi, Badreddine Hassam

**Affiliations:** 1 Service de Dermatologie et Vénérologie, Centre Hospitalier Universitaire IBN SINA, Faculté de Médecine et de Pharmacie, Université Mohammed V, Rabat, Maroc

**Keywords:** Henné, eczema de contact, PPD, Henna, contact eczema, PPD

## Abstract

Henna is used for coloring hair, but also to draw labile skin tattoo. Henna is often mixed with paraphenylenediamine (PPD). We report the case of a female patient presenting with contact eczema due to labile tattoo containing PPD. The study involved a 23-year old young woman with an aesthetic Black Henna tattoo on the back of the hand and on the forearm. This labile tattoo contained PPD. Two days later, the patient developed intensely pruritic, burning erythematovesiculosus and edematous lesions. The lesions involved the tattooed area, corresponding with the original drawing. The lesions were improved using Class 1 of topical corticosteroids (Clobetasol propionate). The patient refused allergy patch testing, so no allergy test was performed. Now, henna is very popular in the western countries. PPD is added to reduce the fixing time or to obtain a darker coloration. It can result in severe systemic reactions. The most frequent allergic reaction is contact dermatitis. Treatment is based on local corticosteroid therapy. A better regulation on labile tattoo, the control of preparations as well as a regular annual information for the general public are essential. The interest of our study lies in highlighting the importance of informing, especially young people, on the risks of labile tattoo.

## Image en médecine

Le henné est un produit utilisé pour colorer les cheveux, mais aussi pour dessiner des tatouages labiles sur la peau. Le henné est souvent mélangé avec de la paraphénylènediamine (PPD). Nous rapportons l'observation d'une patiente qui a présenté un eczéma de contact à un tatouage labile contenant la PPD. Une jeune femme de 23 ans se fait tatouer sur le dos de la main et sur l'avant-bras des dessins esthétiques à base de henné noir. Ce tatouage labile contenait de la PPD. Deux jours plus tard, elle développe des lésions érythématovésiculeuses et œdémateuses très prurigineuses avec une sensation de cuisson. Les lésions siégeaient au site de tatouage et suivaient exactement le dessin initial. Elles étaient améliorées par un traitement à base de dermocorticoïdes topiques de classe 1 (propionate de clobetasol). La patiente refuse l'exploration allergologique par patch-tests et aucun test allergologique n'est effectué. Actuellement, le henné est très en vogue dans les pays occidentaux. La PPD est ajoutée pour diminuer le temps de fixation ou pour obtenir une coloration plus foncée. Elle peut entrainer de graves réactions systémiques. La réaction allergique la plus fréquente est la dermatite de contact. Le traitement repose sur la corticothérapie locale. Une meilleure législation sur la pratique du tatouage temporaire et le contrôle des préparations ainsi qu'une Information régulière annuelle du grand public sont indispensables. L'intérêt de notre observation est de montrer l'importance d'informer surtout les jeunes sur les risques d'un tatouage labile.

**Figure 1:**
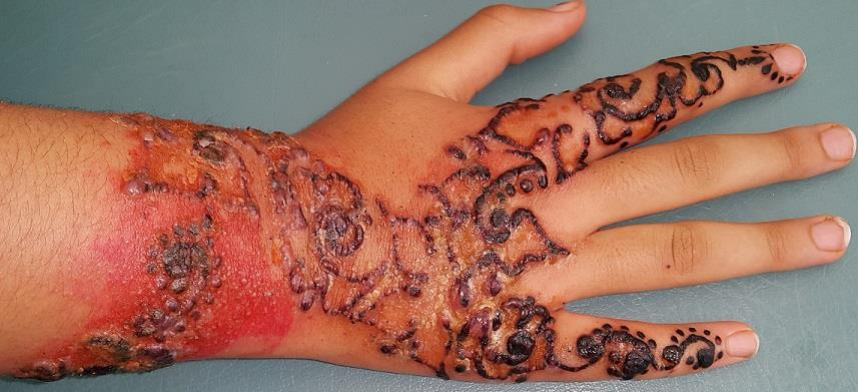
Eczéma bulleux après tatouage éphémère à base de henné noir

